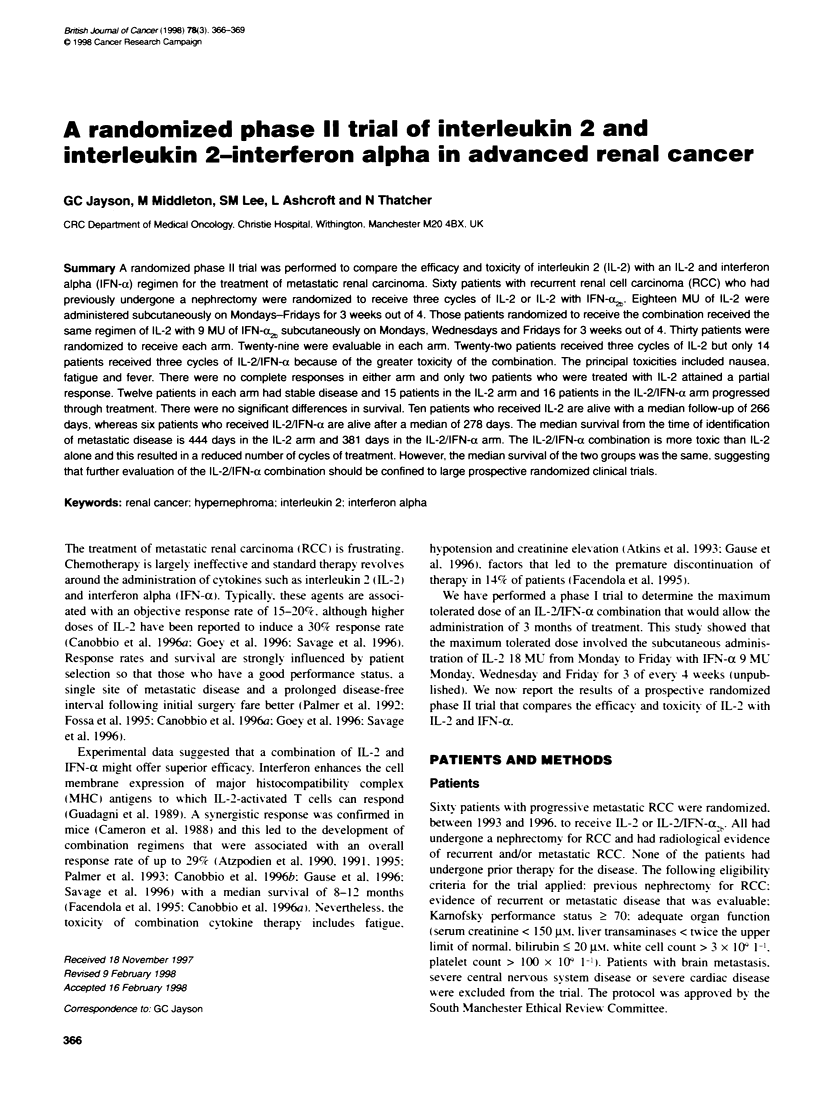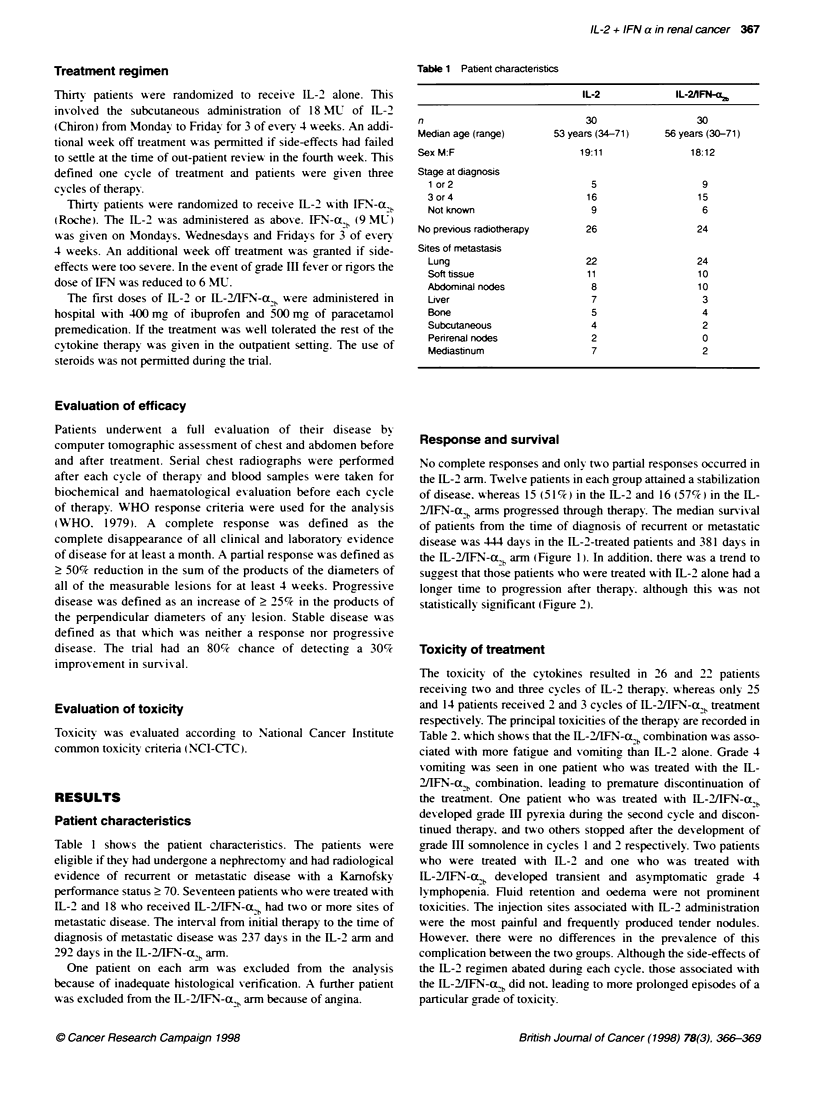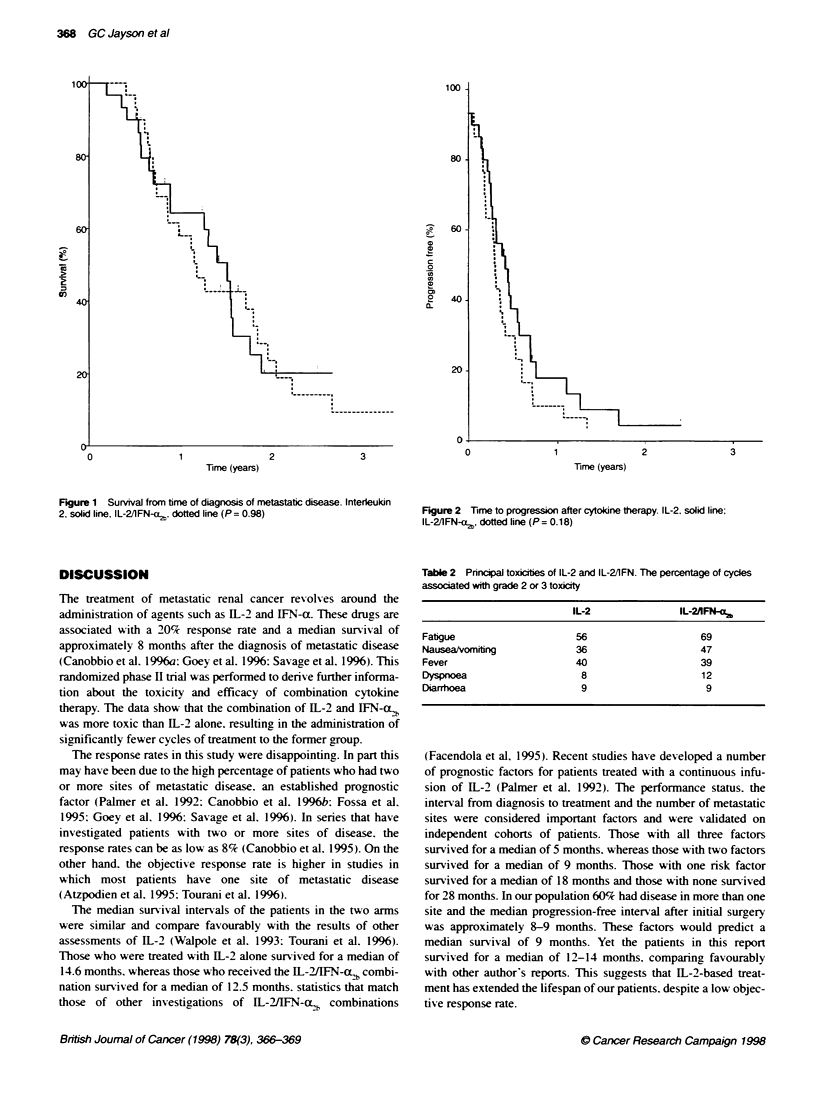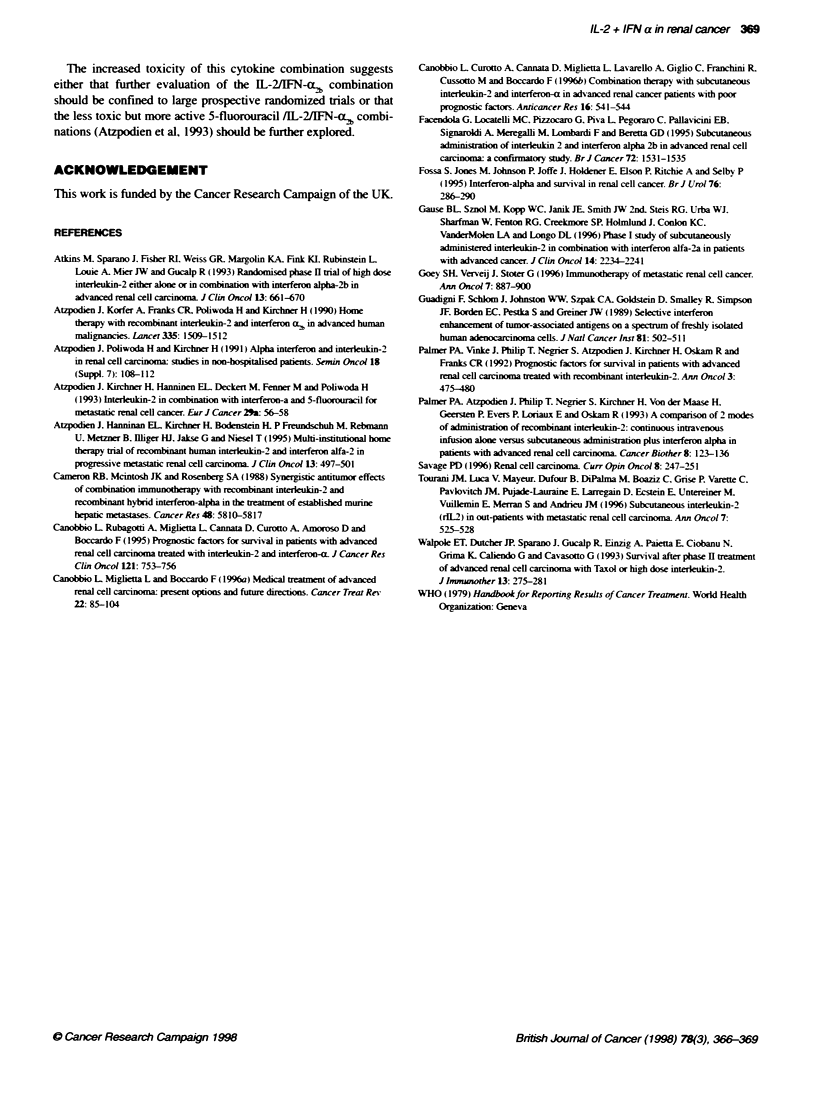# A randomized phase II trial of interleukin 2 and interleukin 2-interferon alpha in advanced renal cancer.

**DOI:** 10.1038/bjc.1998.500

**Published:** 1998-08

**Authors:** G. C. Jayson, M. Middleton, S. M. Lee, L. Ashcroft, N. Thatcher

**Affiliations:** CRC Department of Medical Oncology, Christie Hospital, Withington, Manchester, UK.

## Abstract

A randomized phase II trial was performed to compare the efficacy and toxicity of interleukin 2 (IL-2) with an IL-2 and interferon alpha (IFN-alpha) regimen for the treatment of metastatic renal carcinoma. Sixty patients with recurrent renal cell carcinoma (RCC) who had previously undergone a nephrectomy were randomized to receive three cycles of IL-2 or IL-2 with IFN-alpha2b. Eighteen MU of IL-2 were administered subcutaneously on Mondays-Fridays for 3 weeks out of 4. Those patients randomized to receive the combination received the same regimen of IL-2 with 9 MU of IFN-alpha2b subcutaneously on Mondays, Wednesdays and Fridays for 3 weeks out of 4. Thirty patients were randomized to receive each arm. Twenty-nine were evaluable in each arm. Twenty-two patients received three cycles of IL-2 but only 14 patients received three cycles of IL-2/IFN-alpha because of the greater toxicity of the combination. The principal toxicities included nausea, fatigue and fever. There were no complete responses in either arm and only two patients who were treated with IL-2 attained a partial response. Twelve patients in each arm had stable disease and 15 patients in the IL-2 arm and 16 patients in the IL-2/IFN-alpha arm progressed through treatment. There were no significant differences in survival. Ten patients who received IL-2 are alive with a median follow-up of 266 days, whereas six patients who received IL-2/IFN-alpha are alive after a median of 278 days. The median survival from the time of identification of metastatic disease is 444 days in the IL-2 arm and 381 days in the IL-2/IFN-alpha arm. The IL-2/IFN-alpha combination is more toxic than IL-2 alone and this resulted in a reduced number of cycles of treatment. However, the median survival of the two groups was the same, suggesting that further evaluation of the IL-2/IFN-alpha combination should be confined to large prospective randomized clinical trials.


British Journal of Cancer (1998) 78(3), 366–369
© 1998 Cancer Research Campaign

### A randomized phase II trial of interleukin 2 and interleukin 2–interferon alpha in advanced renal cancer

**GC Jayson, M Middleton, SM Lee, L Ashcroft and N Thatcher**

CRC Department of Medical Oncology, Christie Hospital, Withington, Manchester M20 4BX, UK

**Summary** A randomized phase II trial was performed to compare the efficacy and toxicity of interleukin 2 (IL-2) with an IL-2 and interferon alpha (IFN-α) regimen for the treatment of metastatic renal carcinoma. Sixty patients with recurrent renal cell carcinoma (RCC) who had previously undergone a nephrectomy were randomized to receive three cycles of IL-2 or IL-2 with IFN-$\alpha_{2b}$. Eighteen MU of IL-2 were administered subcutaneously on Mondays–Fridays for 3 weeks out of 4. Those patients randomized to receive the combination received the same regimen of IL-2 with 9 MU of IFN-$\alpha_{2b}$ subcutaneously on Mondays, Wednesdays and Fridays for 3 weeks out of 4. Thirty patients were randomized to receive each arm. Twenty-nine were evaluable in each arm. Twenty-two patients received three cycles of IL-2 but only 14 patients received three cycles of IL-2/IFN-α because of the greater toxicity of the combination. The principal toxicities included nausea, fatigue and fever. There were no complete responses in either arm and only two patients who were treated with IL-2 attained a partial response. Twelve patients in each arm had stable disease and 15 patients in the IL-2 arm and 16 patients in the IL-2/IFN-α arm progressed through treatment. There were no significant differences in survival. Ten patients who received IL-2 are alive with a median follow-up of 266 days, whereas six patients who received IL-2/IFN-α are alive after a median of 278 days. The median survival from the time of identification of metastatic disease is 444 days in the IL-2 arm and 381 days in the IL-2/IFN-α arm. The IL-2/IFN-α combination is more toxic than IL-2 alone and this resulted in a reduced number of cycles of treatment. However, the median survival of the two groups was the same, suggesting that further evaluation of the IL-2/IFN-α combination should be confined to large prospective randomized clinical trials.

**Keywords:** renal cancer; hypernephroma; interleukin 2; interferon alpha

The treatment of metastatic renal carcinoma (RCC) is frustrating. Chemotherapy is largely ineffective and standard therapy revolves around the administration of cytokines such as interleukin 2 (IL-2) and interferon alpha (IFN-α). Typically, these agents are associated with an objective response rate of 15–20%, although higher doses of IL-2 have been reported to induce a 30% response rate (Canobbio et al, 1996*a*; Goey et al, 1996; Savage et al, 1996). Response rates and survival are strongly influenced by patient selection so that those who have a good performance status, a single site of metastatic disease and a prolonged disease-free interval following initial surgery fare better (Palmer et al, 1992; Fossa et al, 1995; Canobbio et al, 1996*a*; Goey et al, 1996; Savage et al, 1996).

Experimental data suggested that a combination of IL-2 and IFN-α might offer superior efficacy. Interferon enhances the cell membrane expression of major histocompatibility complex (MHC) antigens to which IL-2-activated T cells can respond (Guadagni et al, 1989). A synergistic response was confirmed in mice (Cameron et al, 1988) and this led to the development of combination regimens that were associated with an overall response rate of up to 29% (Atzpodien et al, 1990, 1991, 1995; Palmer et al, 1993; Canobbio et al, 1996*b*; Gause et al, 1996; Savage et al, 1996) with a median survival of 8–12 months (Facendola et al, 1995; Canobbio et al, 1996*a*). Nevertheless, the toxicity of combination cytokine therapy includes fatigue, hypotension and creatinine elevation (Atkins et al, 1993; Gause et al, 1996), factors that led to the premature discontinuation of therapy in 14% of patients (Facendola et al, 1995).

We have performed a phase I trial to determine the maximum tolerated dose of an IL-2/IFN-α combination that would allow the administration of 3 months of treatment. This study showed that the maximum tolerated dose involved the subcutaneous administration of IL-2 18 MU from Monday to Friday with IFN-α 9 MU Monday, Wednesday and Friday for 3 of every 4 weeks (unpublished). We now report the results of a prospective randomized phase II trial that compares the efficacy and toxicity of IL-2 with IL-2 and IFN-α.

### PATIENTS AND METHODS

### Patients

Sixty patients with progressive metastatic RCC were randomized, between 1993 and 1996, to receive IL-2 or IL-2/IFN-$\alpha_{2b}$. All had undergone a nephrectomy for RCC and had radiological evidence of recurrent and/or metastatic RCC. None of the patients had undergone prior therapy for the disease. The following eligibility criteria for the trial applied: previous nephrectomy for RCC; evidence of recurrent or metastatic disease that was evaluable; Karnofsky performance status ≥ 70; adequate organ function (serum creatinine < 150 μM, liver transaminases < twice the upper limit of normal, bilirubin ≤ 20 μM, white cell count > 3 × $10^9$ $l^{-1}$, platelet count > 100 × $10^9$ $l^{-1}$). Patients with brain metastasis, severe central nervous system disease or severe cardiac disease were excluded from the trial. The protocol was approved by the South Manchester Ethical Review Committee.

*Received 18 November 1997*
*Revised 9 February 1998*
*Accepted 16 February 1998*

*Correspondence to:* GC Jayson

### Treatment regimen

Thirty patients were randomized to receive IL-2 alone. This involved the subcutaneous administration of 18 MU of IL-2 (Chiron) from Monday to Friday for 3 of every 4 weeks. An additional week off treatment was permitted if side-effects had failed to settle at the time of out-patient review in the fourth week. This defined one cycle of treatment and patients were given three cycles of therapy.

Thirty patients were randomized to receive IL-2 with IFN-$\alpha_{2b}$ (Roche). The IL-2 was administered as above. IFN-$\alpha_{2b}$ (9 MU) was given on Mondays, Wednesdays and Fridays for 3 of every 4 weeks. An additional week off treatment was granted if side-effects were too severe. In the event of grade III fever or rigors the dose of IFN was reduced to 6 MU.

The first doses of IL-2 or IL-2/IFN-$\alpha_{2b}$ were administered in hospital with 400 mg of ibuprofen and 500 mg of paracetamol premedication. If the treatment was well tolerated the rest of the cytokine therapy was given in the outpatient setting. The use of steroids was not permitted during the trial.

### Evaluation of efficacy

Patients underwent a full evaluation of their disease by computer tomographic assessment of chest and abdomen before and after treatment. Serial chest radiographs were performed after each cycle of therapy and blood samples were taken for biochemical and haematological evaluation before each cycle of therapy. WHO response criteria were used for the analysis (WHO, 1979). A complete response was defined as the complete disappearance of all clinical and laboratory evidence of disease for at least a month. A partial response was defined as $\geq$ 50% reduction in the sum of the products of the diameters of all of the measurable lesions for at least 4 weeks. Progressive disease was defined as an increase of $\geq$ 25% in the products of the perpendicular diameters of any lesion. Stable disease was defined as that which was neither a response nor progressive disease. The trial had an 80% chance of detecting a 30% improvement in survival.

### Evaluation of toxicity

Toxicity was evaluated according to National Cancer Institute common toxicity criteria (NCI-CTC).

### RESULTS

### Patient characteristics

Table 1 shows the patient characteristics. The patients were eligible if they had undergone a nephrectomy and had radiological evidence of recurrent or metastatic disease with a Karnofsky performance status $\geq$ 70. Seventeen patients who were treated with IL-2 and 18 who received IL-2/IFN-$\alpha_{2b}$ had two or more sites of metastatic disease. The interval from initial therapy to the time of diagnosis of metastatic disease was 237 days in the IL-2 arm and 292 days in the IL-2/IFN-$\alpha_{2b}$ arm.

One patient on each arm was excluded from the analysis because of inadequate histological verification. A further patient was excluded from the IL-2/IFN-$\alpha_{2b}$ arm because of angina.

**Table 1** Patient characteristics

| | IL-2 | IL-2/IFN-$\alpha_{2b}$ |
|---|---|---|
| *n* | 30 | 30 |
| Median age (range) | 53 years (34–71) | 56 years (30–71) |
| Sex M:F | 19:11 | 18:12 |
| Stage at diagnosis | | |
| 1 or 2 | 5 | 9 |
| 3 or 4 | 16 | 15 |
| Not known | 9 | 6 |
| No previous radiotherapy | 26 | 24 |
| Sites of metastasis | | |
| Lung | 22 | 24 |
| Soft tissue | 11 | 10 |
| Abdominal nodes | 8 | 10 |
| Liver | 7 | 3 |
| Bone | 5 | 4 |
| Subcutaneous | 4 | 2 |
| Perirenal nodes | 2 | 0 |
| Mediastinum | 7 | 2 |

### Response and survival

No complete responses and only two partial responses occurred in the IL-2 arm. Twelve patients in each group attained a stabilization of disease, whereas 15 (51%) in the IL-2 and 16 (57%) in the IL-2/IFN-$\alpha_{2b}$ arms progressed through therapy. The median survival of patients from the time of diagnosis of recurrent or metastatic disease was 444 days in the IL-2-treated patients and 381 days in the IL-2/IFN-$\alpha_{2b}$ arm (Figure 1). In addition, there was a trend to suggest that those patients who were treated with IL-2 alone had a longer time to progression after therapy, although this was not statistically significant (Figure 2).

### Toxicity of treatment

The toxicity of the cytokines resulted in 26 and 22 patients receiving two and three cycles of IL-2 therapy, whereas only 25 and 14 patients received 2 and 3 cycles of IL-2/IFN-$\alpha_{2b}$ treatment respectively. The principal toxicities of the therapy are recorded in Table 2, which shows that the IL-2/IFN-$\alpha_{2b}$ combination was associated with more fatigue and vomiting than IL-2 alone. Grade 4 vomiting was seen in one patient who was treated with the IL-2/IFN-$\alpha_{2b}$ combination, leading to premature discontinuation of the treatment. One patient who was treated with IL-2/IFN-$\alpha_{2b}$ developed grade III pyrexia during the second cycle and discontinued therapy, and two others stopped after the development of grade III somnolence in cycles 1 and 2 respectively. Two patients who were treated with IL-2 and one who was treated with IL-2/IFN-$\alpha_{2b}$ developed transient and asymptomatic grade 4 lymphopenia. Fluid retention and oedema were not prominent toxicities. The injection sites associated with IL-2 administration were the most painful and frequently produced tender nodules. However, there were no differences in the prevalence of this complication between the two groups. Although the side-effects of the IL-2 regimen abated during each cycle, those associated with the IL-2/IFN-$\alpha_{2b}$ did not, leading to more prolonged episodes of a particular grade of toxicity.

© Cancer Research Campaign 1998

**Figure 1** Survival from time of diagnosis of metastatic disease. Interleukin 2, solid line, IL-2/IFN-$\alpha_{2b}$, dotted line ($P$ = 0.98)

**Figure 2** Time to progression after cytokine therapy. IL-2, solid line; IL-2/IFN-$\alpha_{2b}$, dotted line ($P$ = 0.18)

### DISCUSSION

The treatment of metastatic renal cancer revolves around the administration of agents such as IL-2 and IFN-α. These drugs are associated with a 20% response rate and a median survival of approximately 8 months after the diagnosis of metastatic disease (Canobbio et al, 1996*a*; Goey et al, 1996; Savage et al, 1996). This randomized phase II trial was performed to derive further information about the toxicity and efficacy of combination cytokine therapy. The data show that the combination of IL-2 and IFN-$\alpha_{2b}$ was more toxic than IL-2 alone, resulting in the administration of significantly fewer cycles of treatment to the former group.

The response rates in this study were disappointing. In part this may have been due to the high percentage of patients who had two or more sites of metastatic disease, an established prognostic factor (Palmer et al, 1992; Canobbio et al, 1996*b*; Fossa et al, 1995; Goey et al, 1996; Savage et al, 1996). In series that have investigated patients with two or more sites of disease, the response rates can be as low as 8% (Canobbio et al, 1995). On the other hand, the objective response rate is higher in studies in which most patients have one site of metastatic disease (Atzpodien et al, 1995; Tourani et al, 1996).

The median survival intervals of the patients in the two arms were similar and compare favourably with the results of other assessments of IL-2 (Walpole et al, 1993; Tourani et al, 1996). Those who were treated with IL-2 alone survived for a median of 14.6 months, whereas those who received the IL-2/IFN-$\alpha_{2b}$ combination survived for a median of 12.5 months, statistics that match those of other investigations of IL-2/IFN-$\alpha_{2b}$ combinations

**Table 2** Principal toxicities of IL-2 and IL-2/IFN. The percentage of cycles associated with grade 2 or 3 toxicity

| | IL-2 | IL-2/IFN-$\alpha_{2b}$ |
|---|---|---|
| Fatigue | 56 | 69 |
| Nausea/vomiting | 36 | 47 |
| Fever | 40 | 39 |
| Dyspnoea | 8 | 12 |
| Diarrhoea | 9 | 9 |

(Facendola et al, 1995). Recent studies have developed a number of prognostic factors for patients treated with a continuous infusion of IL-2 (Palmer et al, 1992). The performance status, the interval from diagnosis to treatment and the number of metastatic sites were considered important factors and were validated on independent cohorts of patients. Those with all three factors survived for a median of 5 months, whereas those with two factors survived for a median of 9 months. Those with one risk factor survived for a median of 18 months and those with none survived for 28 months. In our population 60% had disease in more than one site and the median progression-free interval after initial surgery was approximately 8–9 months. These factors would predict a median survival of 9 months. Yet the patients in this report survived for a median of 12–14 months, comparing favourably with other author's reports. This suggests that IL-2-based treatment has extended the lifespan of our patients, despite a low objective response rate.

© Cancer Research Campaign 1998

The increased toxicity of this cytokine combination suggests either that further evaluation of the IL-2/IFN-$\alpha_{2b}$ combination should be confined to large prospective randomized trials or that the less toxic but more active 5-fluorouracil /IL-2/IFN-$\alpha_{2b}$ combinations (Atzpodien et al, 1993) should be further explored.

### ACKNOWLEDGEMENT

This work is funded by the Cancer Research Campaign of the UK.

### REFERENCES

Atkins M. Sparano J. Fisher RI. Weiss GR. Margolin KA. Fink KI. Rubinstein L. Louie A. Mier JW and Gucalp R (1993) Randomised phase II trial of high dose interleukin-2 either alone or in combination with interferon alpha-2b in advanced renal cell carcinoma. *J Clin Oncol* **13**: 661–670

Atzpodien J. Korfer A. Franks CR. Poliwoda H and Kirchner H (1990) Home therapy with recombinant interleukin-2 and interferon $\alpha_{2b}$ in advanced human malignancies. *Lancet* **335**: 1509–1512

Atzpodien J. Poliwoda H and Kirchner H (1991) Alpha interferon and interleukin-2 in renal cell carcinoma: studies in non-hospitalised patients. *Semin Oncol* **18** (Suppl. 7): 108–112

Atzpodien J. Kirchner H. Hanninen EL. Deckert M. Fenner M and Poliwoda H (1993) Interleukin-2 in combination with interferon-a and 5-fluorouracil for metastatic renal cell cancer. *Eur J Cancer* **29a**: 56–58

Atzpodien J. Hanninan EL. Kirchner H. Bodenstein H. P Freundschuh M. Rebmann U. Metzner B. Illiger HJ. Jakse G and Niesel T (1995) Multi-institutional home therapy trial of recombinant human interleukin-2 and interferon alfa-2 in progressive metastatic renal cell carcinoma. *J Clin Oncol* **13**: 497–501

Cameron RB. Mcintosh JK and Rosenberg SA (1988) Synergistic antitumor effects of combination immunotherapy with recombinant interleukin-2 and recombinant hybrid interferon-alpha in the treatment of established murine hepatic metastases. *Cancer Res* **48**: 5810–5817

Canobbio L. Rubagotti A. Miglietta L. Cannata D. Curotto A. Amoroso D and Boccardo F (1995) Prognostic factors for survival in patients with advanced renal cell carcinoma treated with interleukin-2 and interferon-α. *J Cancer Res Clin Oncol* **121**: 753–756

Canobbio L. Miglietta L and Boccardo F (1996*a*) Medical treatment of advanced renal cell carcinoma: present options and future directions. *Cancer Treat Rev* **22**: 85–104

Canobbio L. Curotto A. Cannata D. Miglietta L. Lavarello A. Giglio C. Franchini R. Cussotto M and Boccardo F (1996*b*) Combination therapy with subcutaneous interleukin-2 and interferon-α in advanced renal cancer patients with poor prognostic factors. *Anticancer Res* **16**: 541–544

Facendola G. Locatelli MC. Pizzocaro G. Piva L. Pegoraro C. Pallavicini EB. Signaroldi A. Meregalli M. Lombardi F and Beretta GD (1995) Subcutaneous administration of interleukin 2 and interferon alpha 2b in advanced renal cell carcinoma: a confirmatory study. *Br J Cancer* **72**: 1531–1535

Fossa S. Jones M. Johnson P. Joffe J. Holdener E. Elson P. Ritchie A and Selby P (1995) Interferon-alpha and survival in renal cell cancer. *Br J Urol* **76**: 286–290

Gause BL. Sznol M. Kopp WC. Janik JE. Smith JW 2nd. Steis RG. Urba WJ. Sharfman W. Fenton RG. Creekmore SP. Holmlund J. Conlon KC. VanderMolen LA and Longo DL (1996) Phase I study of subcutaneously administered interleukin-2 in combination with interferon alfa-2a in patients with advanced cancer. *J Clin Oncol* **14**: 2234–2241

Goey SH. Verveij J. Stoter G (1996) Immunotherapy of metastatic renal cell cancer. *Ann Oncol* **7**: 887–900

Guadigni F. Schlom J. Johnston WW. Szpak CA. Goldstein D. Smalley R. Simpson JF. Borden EC. Pestka S and Greiner JW (1989) Selective interferon enhancement of tumor-associated antigens on a spectrum of freshly isolated human adenocarcinoma cells. *J Natl Cancer Inst* **81**: 502–511

Palmer PA. Vinke J. Philip T. Negrier S. Atzpodien J. Kirchner H. Oskam R and Franks CR (1992) Prognostic factors for survival in patients with advanced renal cell carcinoma treated with recombinant interleukin-2. *Ann Oncol* **3**: 475–480

Palmer PA. Atzpodien J. Philip T. Negrier S. Kirchner H. Von der Maase H. Geersten P. Evers P. Loriaux E and Oskam R (1993) A comparison of 2 modes of administration of recombinant interleukin-2: continuous intravenous infusion alone versus subcutaneous administration plus interferon alpha in patients with advanced renal cell carcinoma. *Cancer Biother* **8**: 123–136

Savage PD (1996) Renal cell carcinoma. *Curr Opin Oncol* **8**: 247–251

Tourani JM. Luca V. Mayeur. Dufour B. DiPalma M. Boaziz C. Grise P. Varette C. Pavlovitch JM. Pujade-Lauraine E. Larregain D. Ecstein E. Untereiner M. Vuillemin E. Merran S and Andrieu JM (1996) Subcutaneous interleukin-2 (rIL2) in out-patients with metastatic renal cell carcinoma. *Ann Oncol* **7**: 525–528

Walpole ET. Dutcher JP. Sparano J. Gucalp R. Einzig A. Paietta E. Ciobanu N. Grima K. Caliendo G and Cavasotto G (1993) Survival after phase II treatment of advanced renal cell carcinoma with Taxol or high dose interleukin-2. *J Immunother* **13**: 275–281

WHO (1979) *Handbook for Reporting Results of Cancer Treatment*. World Health Organization: Geneva

© Cancer Research Campaign 1998